# Mesenchymal Stromal Cells Are More Effective Than Their Extracellular Vesicles at Reducing Lung Injury Regardless of Acute Respiratory Distress Syndrome Etiology

**DOI:** 10.1155/2019/8262849

**Published:** 2019-08-21

**Authors:** Johnatas D. Silva, Ligia L. de Castro, Cassia L. Braga, Gisele P. Oliveira, Stefano A. Trivelin, Carlos M. Barbosa-Junior, Marcelo M. Morales, Claudia C. dos Santos, Daniel J. Weiss, Miquéias Lopes-Pacheco, Fernanda F. Cruz, Patricia R. M. Rocco

**Affiliations:** ^1^Laboratory of Pulmonary Investigation, Carlos Chagas Filho Institute of Biophysics, Federal University of Rio de Janeiro, Rio de Janeiro, Brazil; ^2^National Institute of Science and Technology for Regenerative Medicine, Rio de Janeiro, Brazil; ^3^Laboratory of Cellular and Molecular Physiology, Carlos Chagas Filho Institute of Biophysics, Federal University of Rio de Janeiro, Rio de Janeiro, Brazil; ^4^Interdepartmental Division of Critical Care, Keenan Research Centre for Biomedical Science and Institute of Medical Sciences, Faculty of Medicine, University of Toronto, Toronto, Canada; ^5^Division of Pulmonary Disease and Critical Care Medicine, Department of Medicine, University of Vermont, Burlington, Vermont, USA

## Abstract

Although mesenchymal stromal cells (MSCs) have demonstrated beneficial effects on experimental acute respiratory distress syndrome (ARDS), preconditioning may be required to potentiate their therapeutic effects. Additionally, administration of cell-free products, such as extracellular vesicles (EVs) obtained from MSC-conditioned media, might be as effective as MSCs. In this study, we comparatively evaluated the effects of MSCs, preconditioned or not with serum collected from mice with pulmonary or extrapulmonary ARDS (ARDSp and ARDSexp, respectively), and the EVs derived from these cells on lung inflammation and remodeling in ARDSp and ARDSexp mice. Administration of MSCs (preconditioned or not), but not their EVs, reduced static lung elastance, interstitial edema, and collagen fiber content in both ARDSp and ARDSexp. Although MSCs and EVs reduced alveolar collapse and neutrophil cell counts in lung tissue, therapeutic responses were superior in mice receiving MSCs, regardless of preconditioning. Despite higher total cell, macrophage, and neutrophil counts in bronchoalveolar lavage fluid in ARDSp than ARDSexp, MSCs and EVs (preconditioned or not) led to a similar decrease. In ARDSp, both MSCs and EVs, regardless of preconditioning, reduced levels of tumor necrosis factor- (TNF-) *α*, interleukin-6, keratinocyte chemoattractant (KC), vascular endothelial growth factor (VEGF), and transforming growth factor- (TGF-) *β* in lung homogenates. In ARDSexp, TNF-*α*, interleukin-6, and KC levels were reduced by MSCs and EVs, preconditioned or not; only MSCs reduced VEGF levels, while TGF-*β* levels were similarly increased in ARDSexp treated either with saline, MSCs, or EVs, regardless of preconditioning. In conclusion, MSCs yielded greater overall improvement in ARDS in comparison to EVs derived from the same number of cells and regardless of the preconditioning status. However, the effects of MSCs and EVs differed according to ARDS etiology.

## 1. Introduction

Despite recent advances in supportive care for acute respiratory distress syndrome (ARDS) patients, mortality remains high [1, 2] and those who survive usually face long-term morbidity [3]. Furthermore, several pharmacological approaches have failed to improve clinical outcomes [4]. Therefore, more effective therapeutic approaches for ARDS are required.

Bone marrow-derived mesenchymal stromal cells (MSCs) have been shown to promote immunomodulatory effects by secreting trophic factors [5–8]. Both systemic administration and intratracheal administration of MSCs mitigated pulmonary and systemic inflammation as well as enhanced bacterial clearance, resulting in lower mortality in different models of ARDS [9–12]. Nevertheless, some theoretical safety concerns remain regarding the administration of high doses of MSCs, leading to a potential risk of pulmonary embolism [13]. Accordingly, administration of cell-free products, such as extracellular vesicles (EVs) obtained from MSC-conditioned media, might offer an alternative with similar therapeutic effects on the inflammatory processes in ARDS, without the inherent challenges of using live cells [14–17]. However, the impact of EVs on lung fibrosis, which is an important determinant of ARDS patient outcome, has not yet been specifically investigated and further studies are needed to closely compare the effects of MSCs and their EVs.

Additionally, recent studies have suggested that the anti-inflammatory actions of MSCs can be enhanced by conditioning them prior to administration [18–20]. This reflects the ability of MSCs to respond to different injured microenvironments through differential stimulation of Toll-like receptors and other damage receptors. However, only limited data exist regarding the effects of MSCs preconditioned with single agents, such as eicosapentaenoic acid, or serum obtained from animals with experimentally induced lung injury [18, 21]. Since ARDS pathophysiology may differ according to the type of primary insult, resulting in the activation of different inflammatory mechanisms [22], we hypothesized that serum from mice with experimental endotoxin-induced pulmonary and extrapulmonary ARDS (ARDSp and ARDSexp, respectively) may differently impact on MSCs and their derived EVs. To address the therapeutic potential of “parent” MSCs as compared to their EVs and the effects of preconditioning with biologically relevant specimens, this study is aimed at comparing the impact of systemic administration of MSCs, preconditioned or not with serum from ARDSp and ARDSexp animals and their derived EVs. Endpoints of interest included lung mechanics, histology, total and differential cell counts in lung tissue and bronchoalveolar lavage fluid, and protein levels of selected mediators. Furthermore, the effects of conditioned media and EVs of naïve and preconditioned MSCs on macrophage-produced mediators were investigated. The effects of serum exposure on protein concentrations in MSC-derived EVs were also assessed.

## 2. Material and Methods

### 2.1. Ethics Statement

This study was approved by the Animal Care and Use Committee of the Health Sciences Center, Federal University of Rio de Janeiro (CEUA 020/2017). All animals received humane care in compliance with the “Principles of Laboratory Animal Care” formulated by the National Society for Medical Research and the U.S. National Research Council *Guide for the Care and Use of Laboratory Animals*. The present study followed the ARRIVE guidelines for reporting of animal research [23]. Animals were housed in standard laboratory cages (12 h light/dark cycles, temperature 23 ± 1°C), three in each cage, with access to food and water *ad libitum*.

### 2.2. Animal Preparation and Experimental Protocol

**Figure 1:**
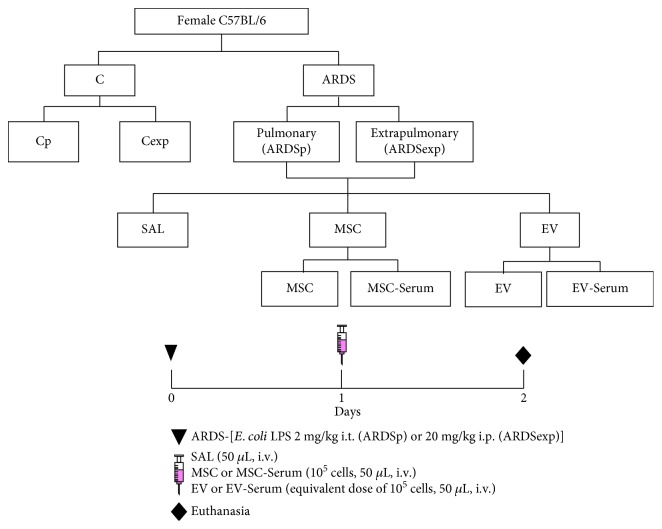
Schematic flow chart and timeline of study design. ARDS was induced by administration of *Escherichia coli* lipopolysaccharide intratracheally (ARDSp) or intraperitoneally (ARDSexp). Control mice (C) received saline solution intratracheally (Cp) or intraperitoneally (Cexp). After 24 h, ARDSp and ARDSexp animals were further randomized to receive saline (50 *μ*L, SAL), bone marrow-derived MSCs (10^5^, 50 *μ*L), or EVs (10^5^, 50 *μ*L), stimulated (MSC serum, EV serum) or not with serum obtained from ARDSp or ARDSexp animals. All data were analyzed on day 2.

All assessments were performed in blinded fashion. A total of 188 C57BL/6 mice (180 females and 8 males, weight 20–25 g, age 8–10 weeks) were used: 96 females for assessment of lung mechanics and histology, 72 females for analysis of bronchoalveolar lavage fluid (BALF), 8 males as cell donors, and 12 females for *in vitro* analysis of the alveolar macrophage phenotype. ARDS was induced in female mice by administering *Escherichia coli* lipopolysaccharide (LPS) (serotype O55:B5, LPS-B5 Ultrapure: TLR4 agonist; InvivoGen, San Diego, CA, USA) intratracheally (2 mg·kg^−1^, ARDSp) or intraperitoneally (20 mg·kg^−1^, ARDSexp). In control (C) groups, sterile saline solution was administered intratracheally (50 *μ*L, Cp) or intraperitoneally (500 *μ*L, Cexp) instead. On the next day, ARDS mice were further randomized into subgroups to receive sterile saline solution (50 *μ*L), bone marrow-derived MSCs stimulated or not with serum (MSC or MSC serum, 10^5^ cells in 50 *μ*L of saline), or EVs obtained from these MSCs (EV or EV serum), all administered via the jugular vein (Figure 1). The total amount of EVs administered was adjusted to correspond to the concentration released by 10^5^ cells. Additionally, 12 female mice were used for collection of alveolar macrophages after exposure to the Cp, Cexp, ARDSp, and ARDSexp protocols. Twenty-four hours after endotoxin administration, serum was collected, pooled, and stored at −80°C until being used to precondition MSCs *in vitro*.

### 2.3. MSC Isolation and Culture Conditions

Male C57BL/6 mice (*n* = 8) were anesthetized with intravenous ketamine (25 mg·kg^−1^) and xylazine (2 mg·kg^−1^) and used as cell donors. Bone marrow cells were obtained from femurs and tibias. After isolation, bone marrow-derived cells were cultured (37°C, 5% CO_2_ in humidified atmosphere) with Iscove's Modified Dulbecco's Medium (IMDM) (Invitrogen, CA) containing 15 mM HEPES (Sigma, MO), 15% inactivated fetal bovine serum (FBS) (Invitrogen, CA), 100 units·mL^−1^ penicillin, and 100 mg·mL^−1^ streptomycin antibiotic solution (P/S; Gibco, NM). On day 3 of culture, the medium was changed and nonadherent cells were removed. Adherent cells exhibited similar proliferation rates. Upon reaching ~80% confluence, they were passaged with 0.05% trypsin-EDTA solution (Gibco, NM) and then maintained in IMDM with 10% FBS and antibiotic solution. MSCs were gradually cryopreserved at –80°C in a concentration of 1 × 10^6^ cells in 1.8 mL of freezing solution containing 50% supplemented IMDM, 40% FBS, and 10% dimethyl sulfoxide (Sigma-Aldrich, St. Louis, MO). Immediately before experimental use, cells were thawed and washed in sterile saline. Cell viability, density, and final concentration (1 × 10^5^ viable cells per 50 *μ*L of saline) were then determined by trypan blue exclusion and by counting in a hemocytometer [17]. At the third passage, approximately 10 million cells were characterized as MSCs through flow cytometry and by inducing differentiation into osteoblasts and chondroblasts, as previously described [7].

### 2.4. MSC Preconditioning with Serum from ARDSp and ARDSexp Animals

MSCs were cultured in 12-well plates (10^5^ cells/well) using high-glucose Dulbecco's Modified Eagle's Medium (DMEM) supplemented with 10% FBS, P/S, and 2 mM L-glutamine (Invitrogen, Life Technologies, Grand Island, NY, USA). MSCs were exposed or not to serum (10% *v*/*v*) from ARDSp and ARDSexp mice for 24 hours. The concentration of 10% *v*/*v* was based on pilot studies and in a previous study by our group conducted in another experimental model [21]. Briefly, MSCs were stimulated with a pool of serum obtained from five CTRL, five ARDSp, and five ARDSexp mice using a concentration curve (0%, 0.25%, 0.5%, 0.75%, 1%, 10%, 20%, 30%, 40%, and 50%) in DMEM supplemented with 10% FBS, P/S, and 2 mM l-glutamine. Concentrations of cytokines and growth factors produced by the cells before and after activation were measured; the 10% concentration was found to be most effective at modulating the MSC secretome.

### 2.5. EV Extraction and Characterization

After 48 hours of FBS deprivation, EVs were obtained from the supernatant of MSCs, as previously described by Zhu and colleagues [15]. Briefly, conditioned media from MSCs of healthy animals were maintained with regular medium or exposed to serum obtained from ARDSp or ARDSexp animals for 24 h and then centrifuged at 3000 g for 20 minutes to remove cellular debris. Thereafter, ultracentrifugation at 100000 g (Beckman Coulter Optima L-100XP Ultracentrifuge, rotor RW 70Ti; Beckman Coulter, Brea, CA) was performed for 1 hour at 4°C to sediment the EVs, which were then washed in saline and subjected to a second round of ultracentrifugation at 100000 g for 1 hour [15]. EVs were resuspended in saline according to the final cell count of MSCs and stored at −80°C until further use.

The total protein content of the EV fraction was quantified by Bradford's assay to ensure that the same amount of EVs would be administered to all animals. Instead of using protein concentration, the dose of EVs was based on the final MSC count which generated the conditioned medium, to allow comparison of findings with ARDS experiments using preconditioned or nonpreconditioned MSCs. The viability of the serum-starved MSCs was >92% at 48 h before EV isolation.

The intensity and hydrodynamic diameter of EVs were measured by dynamic light scattering in a Zetasizer Nano ZS90 system (Malvern Instruments Ltd., Malvern, UK). Forty-eight hours after FBS deprivation, MSCs were fixed in 2.5% glutaraldehyde in 0.1 M sodium cacodylate buffer (pH 7.2) for 2 hours and washed twice with cacodylate buffer. Immediately thereafter, postfixation with OsO_4_ and FeCNK solution (1 : 1) was performed for 45 min, followed by dehydration in a graded ethanol series for 10 minutes at each concentration (30%, 50%, 70%, 90%, 100%, and the latter three times). After critical-point drying, the coverslips were analyzed and images were acquired in a FEI Quanta 250 scanning electron microscope (FEI, Hillsboro, OR, USA).

The absolute size distribution and concentration of EVs were evaluated using nanoparticle tracking analysis (NanoSight NS300, Malvern Instruments Ltd., Malvern, UK). The analysis settings were optimized using filtered PBS as control and kept constant between samples. The NTA measurement conditions were as follows: three measurements per sample (30 s/measurement), temperature 25°C, viscosity 0.9 cP, and 25 frames per second. Each video was analyzed to give the mean, mode, median, and estimated concentration for each particle size. The samples were diluted to obtain the right number of particles (1 × 10^5^ particles/500 *μ*L) proportional to the number of MSCs administered to the animals during *in vivo* experiments.

### 2.6. Lung Mechanics

Twenty-four hours after SAL, MSC, or EV administration, the animals were sedated (diazepam 1 mg·kg^−1^ intraperitoneally), anesthetized (thiopental sodium 20 mg·kg^−1^ intraperitoneally), tracheotomized, paralyzed (vecuronium bromide, 0.005 mg·kg^−1^ intravenously), and ventilated using a constant-flow ventilator (Samay VR15; Universidad de la Republica, Montevideo, Uruguay) with the following settings: respiratory rate (RR) 100 breaths per minute, tidal volume (*V*_T_) 0.2 mL, and fraction of inspired oxygen (FiO_2_) 0.21. The anterior chest wall was surgically removed, and a positive end-expiratory pressure (PEEP) of 2 cmH_2_O was applied. Airflow and tracheal pressure (Ptr) were measured [11, 24]. Lung mechanics were analyzed by the end-inflation occlusion method [24]. In an open chest preparation, Ptr reflects transpulmonary pressure (PL). Briefly, after end-inspiratory occlusion, there is an initial rapid drop in PL from the preocclusion value (Δ*P*1, *L*) down to an inflection point, followed by a slow pressure decay (Δ*P*2, *L*), until a plateau is reached. This plateau corresponds to the elastic recoil pressure of the lung (Pel). Static lung elastance (Est, *L*) was determined by dividing Pel by *V*_T_. Δ*P*1, *L* selectively reflects the pressure used to overcome the airway resistance. Δ*P*2, *L* reproduces the pressure spent by stress relaxation or the viscoelastic properties of the lung, together with a small contribution from *pendelluft*. Lung mechanics measurements were performed 10 times in each animal [11, 25]. All data were analyzed using ANADAT software (RHT-InfoData Inc., Montreal, Quebec, Canada).

### 2.7. Lung Histology

Soon after determination of lung mechanics, laparotomy was performed and heparin (1000 IU) was injected intravenously. The trachea was clamped at end-expiration (PEEP = 2 cmH_2_O), and the abdominal aorta and vena cava were sectioned to cause death by exsanguination. The right lung was removed, fixed in 4% buffered formalin, and embedded in paraffin. Slices (4 *μ*m thick) were mounted on glass slides and stained with hematoxylin and eosin for morphometric analysis. The volume fraction of collapsed and normal pulmonary areas, as well as the number of neutrophils in lung tissue, were determined by the point counting technique at a magnification of ×200 and ×1000, respectively, across 10 random, noncoincident microscopic fields [25, 26]. Collagen fiber was quantified in the alveolar septa by the Picrosirius-polarization method [11, 27]. For quantification of interstitial edema, 10 arteries were transversely sectioned. The number of points falling on areas of perivascular edema and the number of intercepts between the lines of the integrating eyepiece and the basement membrane of the vessels were counted at a magnification of ×400. The interstitial perivascular edema index was calculated by the number of points per number of intercepts, as described elsewhere [28].

### 2.8. Bronchoalveolar Lavage Fluid (BALF) Cellularity and Total Protein Content

Briefly, a separate cohort of mice underwent euthanasia at the end of the study period. Thereafter, the trachea was cannulated and the lung was lavaged three times with 0.4 mL total volume of saline solution containing ethylenediamine tetraacetic acid (10 mM). BALF was centrifuged at 4°C for 10 min at 400 g and the cell pellet was resuspended in saline for further total leukocyte counting in a Neubauer chamber under light microscopy, after diluting the samples in Türk solution (2% acetic acid). Differential cell count was performed in cytospin smears stained by the May-Grünwald-Giemsa method, as previously described [25, 29]. Furthermore, the total protein content in the BALF supernatant was quantified by Bradford's reagent (Sigma-Aldrich, St. Louis, MO, USA).

### 2.9. Enzyme-Linked Immunosorbent Assay (ELISA)

For protein isolation, the right lobes of the lungs were frozen in liquid nitrogen and kept at −80°C until analysis. Lung tissue was homogenized in lysis buffer (PBS 1x, Triton X 0.01%, 1x Roche protease inhibitor cocktail (Roche Diagnostics, Mannheim, Germany)) using a glass Potter homogenizer with a Teflon piston. The total amount of biomarkers was quantified according to the manufacturer's protocol and normalized to the total content of protein as quantified by Bradford's reagent (Sigma-Aldrich, St. Louis, MO, USA). Protein levels of tumor necrosis factor- (TNF-) *α*, IL-6, IL-10, keratinocyte chemoattractant (KC), vascular endothelial growth factor (VEGF), and transforming growth factor- (TGF-) *β* were quantified in lung homogenate with ELISA kits, in accordance with the manufacturer's instructions.

### 2.10. *In Vitro* Analysis of Mediator Production in Alveolar Macrophages

Alveolar macrophages were obtained from the BALF of Cp, Cexp, ARDSp, and ARDSexp mice [30]. BALF of three mice per group was pooled to obtain enough alveolar macrophages for analysis. Experiments were performed in triplicate. BALF was centrifuged at 300 g for 10 min, and the cellular *pellet* was washed with saline, resuspended in red blood cell lysis buffer (8.3 g NH_4_Cl, 1 g KHCO_3_, 1.8 mL 5% EDTA in 1 L distilled water) for 5 min at room temperature, and centrifuged again at 300 g for 10 min. The pelleted cells were resuspended and cultured in a 12-well culture plate at 37°C with 5% CO_2_ at a concentration of 10^5^ cells per well in 1 mL RPMI 1640 medium (Sigma Chemical Co., St. Louis, MO) supplemented with 10% FBS, 1 mM pyruvate, 1% nonessential amino acids, 14 mM glucose, 17.9 mM NaHCO_3_, 10 mM HEPES, 100 U/mL penicillin, and 0.1 mg/mL streptomycin. After 2 hours of incubation, nonadherent cells were washed off with saline and the medium was refreshed. Alveolar macrophages were stimulated with conditioned media obtained from MSCs stimulated or not with serum of Cp, Cexp, ARDSp, and ARDSexp mice for an additional 24 hours. Alveolar macrophages were then washed with sterile saline, harvested from the culture plates, and pelleted by centrifugation (600 g for 5 min). RT-qPCR was performed as previously described [21]. The relative level of each gene was calculated as the ratio of the study gene to the housekeeping gene (*36B4*) and given as the fold change relative to the C group (alveolar macrophages from the Cp or Cexp group). Then, mRNA expression for the following genes was analyzed: inducible nitric oxide synthase (iNOS), IL-6, and IL-*β* (proinflammatory markers) and arginase-2, IL-10, and TGF-*β* (anti-inflammatory markers). The sequence of each PCR primer is provided in Supplementary [Supplementary-material supplementary-material-1].

### 2.11. Statistical Analysis

The sample size was calculated to allow detection of the differences in Est, *L* after MSC therapy in ARDSp and ARDSexp animals, based on the previous work from our group [11]. A sample size of 6 animals per group would provide the appropriate power (1 − *β* = 0.8) to identify statistically significant differences in Est, *L* (adjusted *α* = 0.025 for two comparisons), taking into account an effect size *d* = 2.0, a two-sided *t*-test, and a sample size ratio = 1 (G∗Power 3.1.9.2, University of Düsseldorf, Düsseldorf, Germany).

Data were tested for normality using the Kolmogorov-Smirnov test with Lilliefors' correction, while the Levene median test was used to evaluate the homogeneity of variances. If both conditions were satisfied, differences among groups at each ARDS etiology were determined with one-way ANOVA test followed by Tukey's test. Molecular biology variables were assessed with the Kruskal-Wallis test followed by Dunn's test. Parametric data were expressed as mean ± SD, while nonparametric data were expressed as median (interquartile range). Statistical analyses were carried out in GraphPad Prism 6.07 (GraphPad Software, La Jolla, CA, USA). Significance was established at *p* < 0.05.

## 3. Results

### 3.1. Serum from ARDSp and ARDSexp Mice Did Not Affect Protein Concentration on MSC-Secreted EVs

MSCs cultured under regular conditions demonstrated the presence of both exosomes and microvesicles on the cell surface (Supplementary Fig. [Supplementary-material supplementary-material-1]-[Supplementary-material supplementary-material-1]). MSCs preconditioned with serum from either ARDSp or ARDSexp animals also demonstrated formation of exosomes and microvesicles on MSC surfaces (Supplementary Fig. [Supplementary-material supplementary-material-1]-[Supplementary-material supplementary-material-1]). Nonetheless, no significant difference was observed in protein concentration as evaluated by the Bradford assay among groups (Supplementary Fig. [Supplementary-material supplementary-material-1]). We found increases in particle size and EV concentration after MSC stimulation with serum from ARDSp and ARDSexp animals (Supplementary Fig. [Supplementary-material supplementary-material-1]).

### 3.2. MSCs Were More Effective at Reducing Lung Morphometric Abnormalities, Inflammation, and Collagen Fiber Content Than Their EVs, Regardless of Preconditioning Status

**Figure 2:**
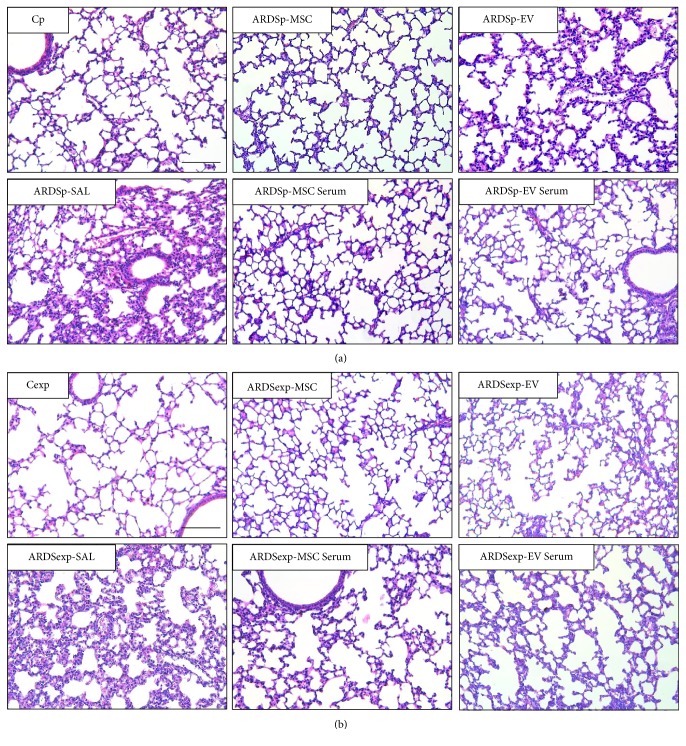
Lung histology. Representative photomicrographs of lung parenchyma stained with hematoxylin and eosin from (a) pulmonary ARDS (ARDSp) and (b) extrapulmonary ARDS (ARDSexp) animals. ARDS was induced by administration of *Escherichia coli* lipopolysaccharide intratracheally (ARDSp) or intraperitoneally (ARDSexp). Control mice (C) received saline solution intratracheally (Cp) or intraperitoneally (Cexp). After 24 h, ARDSp and ARDSexp animals were further randomized to receive saline (50 *μ*L, SAL), bone marrow-derived MSCs (10^5^, 50 *μ*L), or EVs (10^5^, 50 *μ*L), stimulated (MSC serum, EV serum) or not (MSCs, EVs) with serum obtained from ARDSp or ARDSexp animals.

ARDSp-SAL and ARDSexp-SAL animals exhibited an increased fraction area of alveolar collapse, neutrophil cell count, interstitial edema, and collagen fiber content compared to Cp and Cexp animals, respectively (Table 1, Figure 2).

In both the ARDSp and ARDSexp groups, alveolar collapse and neutrophil counts were reduced after either MSC or EV administration, regardless of preconditioning status; however, MSCs induced a better response than EVs. Furthermore, MSCs, but not EVs, reduced interstitial edema and collagen fiber content in ARDSp and ARDSexp animals, regardless of preconditioning status (Table 1, Figure 2).

### 3.3. Administration of MSCs or Their EVs Led to Reductions in BALF Cellularity and Total Protein Content, Regardless of Preconditioning Status

**Figure 3:**
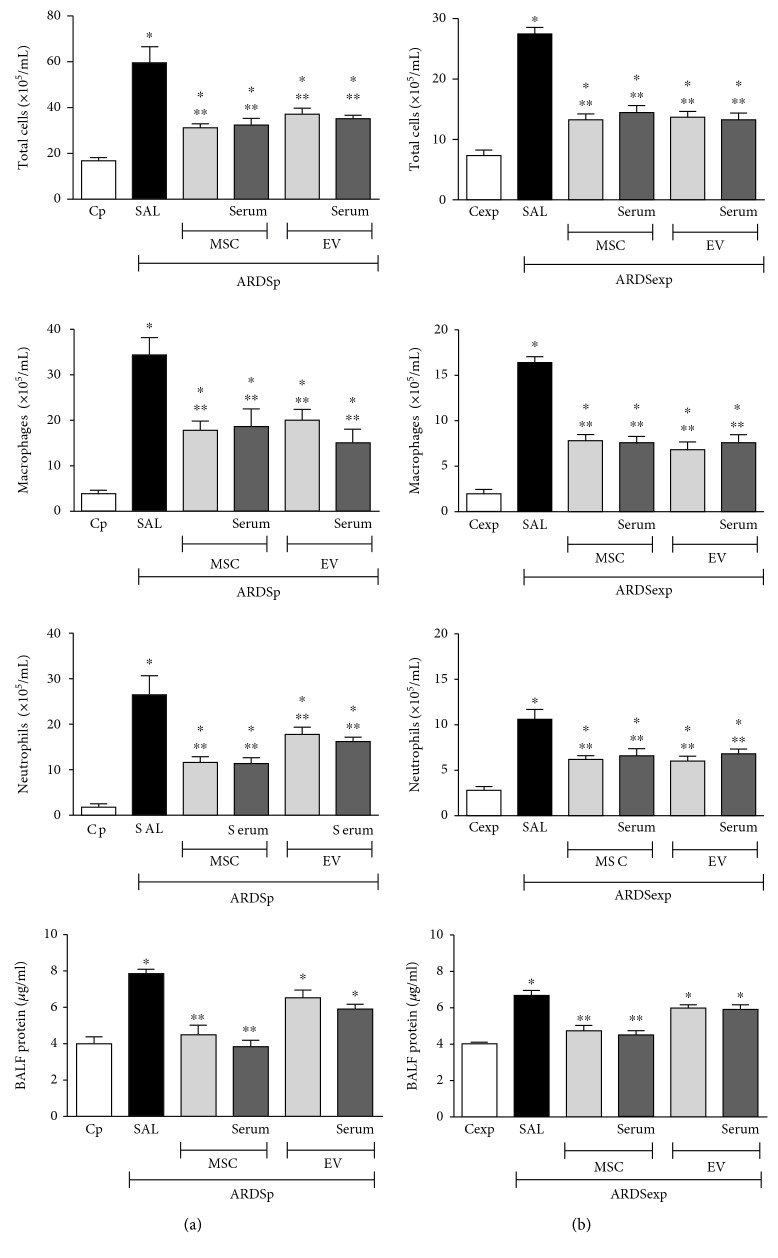
Total and differential cell counts, as well as protein content, in bronchoalveolar lavage fluid in pulmonary ARDS (ARDSp) (a) and extrapulmonary ARDS (ARDSexp) (b) animals. ARDS was induced by administration of *Escherichia coli* lipopolysaccharide intratracheally (ARDSp) or intraperitoneally (ARDSexp). Control mice (C) received saline solution intratracheally (Cp) or intraperitoneally (Cexp). After 24 h, ARDSp and ARDSexp animals were further randomized to receive saline (50 *μ*L, SAL), bone marrow-derived MSCs (10^5^, 50 *μ*L), or EVs (10^5^, 50 *μ*L), stimulated or not with serum (MSCs, EVs, MSC serum, and EV serum) obtained from ARDSp or ARDSexp animals. Values were expressed as mean ± standard deviation of 6 animals per group. ^∗^Significantly different from the corresponding C group (*p* < 0.05). ^∗∗^Significantly different from the corresponding ARDS group (*p* < 0.05).

The ARDSp-SAL and ARDSexp-SAL groups demonstrated an increase in total and differential cell counts and total protein content in BALF compared to Cp and Cexp, respectively (Figure 3). Regardless of preconditioning status, MSCs and EVs were able to comparably reduce the number of total cells, macrophages, and neutrophils as well as total protein content in the BALF (Figure 3).

### 3.4. Administration of MSCs, but Not EVs, Was Effective at Improving Lung Mechanics, Regardless of Serum Stimulation

**Figure 4:**
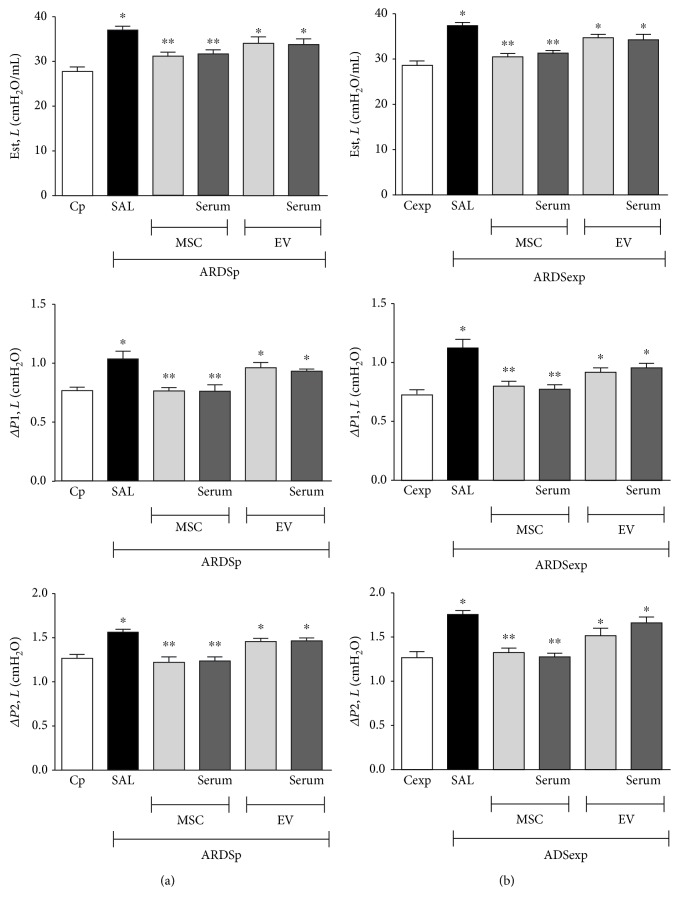
Lung mechanics. Static lung elastance (Est, *L*) and resistive (Δ*P*1, *L*) and viscoelastic (Δ*P*2, *L*) pressures in animals with experimental pulmonary ARDS (ARDSp) (a) and extrapulmonary ARDS (ARDSexp) (b). ARDS was induced by administration of *Escherichia coli* lipopolysaccharide intratracheally (ARDSp) or intraperitoneally (ARDSexp). Control mice (C) received saline solution intratracheally (Cp) or intraperitoneally (Cexp). After 24 h, ARDSp and ARDSexp animals were further randomized to receive saline (50 *μ*L, SAL), bone marrow-derived MSCs (10^5^, 50 *μ*L), or EVs (10^5^, 50 *μ*L), stimulated (MSC serum, EV serum) or not (MSCs, EVs) with serum obtained from ARDSp or ARDSexp animals. Values were expressed as mean + standard deviation of 6 animals per group. ^∗^Significantly different from the corresponding C group (*p* < 0.05). ^∗∗^Significantly different from the corresponding ARDS group (*p* < 0.05).

ARDSp-SAL and ARDSexp-SAL animals demonstrated increased Est, *L* (*p* = 0.01 and *p* < 0.0001), Δ*P*1, *L* (*p* = 0.004 and *p* = 0.007), and Δ*P*2, *L* (*p* = 0.0002 and *p* < 0.0001) (Figure 4) compared to Cp and Cexp animals, respectively. In both the ARDSp and ARDSexp groups, MSCs, but not EVs, were effective at reducing Est, *L*, Δ*P*1, *L*, and Δ*P*2, *L* regardless of preconditioning status (Figure 4).

### 3.5. Administration of MSCs or Their EVs Differentially Modulated Protein Levels of Biomarkers in Lung Tissue Homogenate Depending on ARDS Etiology

**Figure 5:**
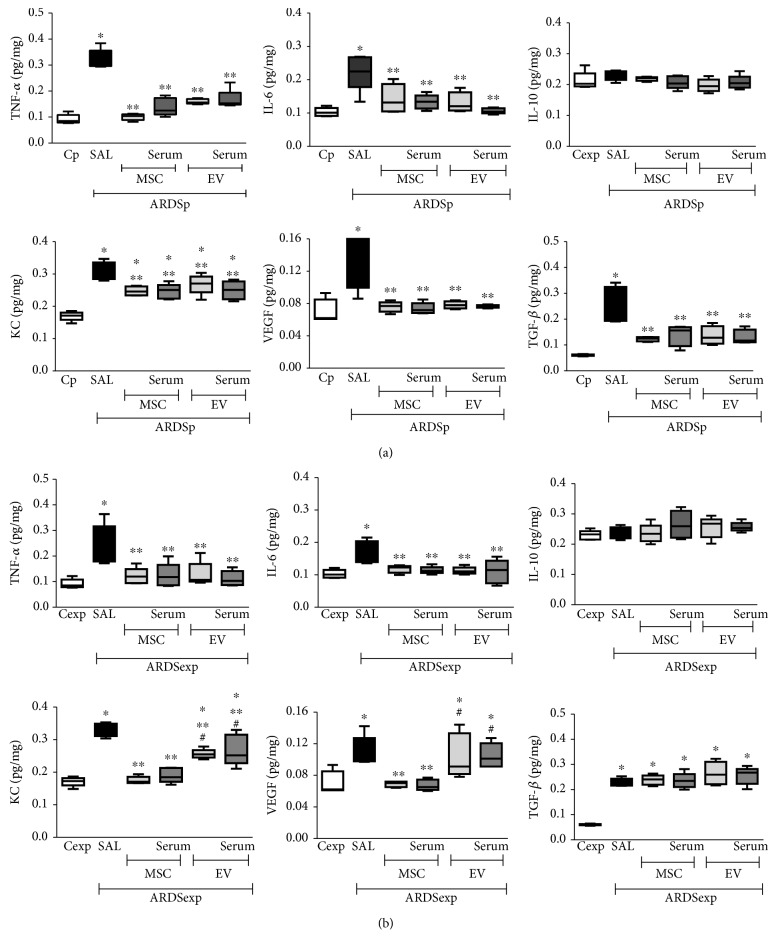
Protein levels of mediators in lung tissue. Protein levels of tumor necrosis factor- (TNF-) *α*, interleukin- (IL-) 6, IL-10, keratinocyte chemoattractant (KC) (a murine IL-8 homolog), vascular endothelial growth factor (VEGF), and transforming growth factor- (TGF-) *β* in lung tissue homogenate from (a) pulmonary ARDS (ARDSp) and (b) extrapulmonary ARDS (ARDSexp) animals. ARDS was induced by administration of *Escherichia coli* lipopolysaccharide intratracheally (ARDSp) or intraperitoneally (ARDSexp). Control mice (C) received saline solution intratracheally (Cp) or intraperitoneally (Cexp). After 24 h, ARDSp and ARDSexp animals were further randomized to receive saline (50 *μ*L, SAL), bone marrow-derived MSCs (10^5^, 50 *μ*L), or EVs (10^5^, 50 *μ*L), stimulated (MSC serum, EV serum) or not with serum (MSCs, EVs) obtained from ARDSp or ARDSexp animals. Boxes show the interquartile (P25-P75) range, whiskers denote the range (minimum-maximum), and the horizontal line represents the median of animals per group. ^∗^Significantly different from the corresponding C group (*p* < 0.05). ^∗∗^Significantly different from the corresponding ARDS group (*p* < 0.05). ^#^Significantly different from the corresponding MSC group (*p* < 0.05).

TNF-*α*, IL-6, KC, VEGF, and TGF-*β* protein levels were increased in lung tissues from ARDSp (Figure 5(a)) and ARDSexp (Figure 5(b)) animals treated with saline compared to Cp and Cexp, respectively. IL-10 levels were similar among groups in both ARDSp (Figure 5(a)) and ARDSexp (Figure 5(b)).

In ARDSp, both MSCs and EVs comparably reduced protein levels of TNF-*α*, IL-6, KC, VEGF, and TGF-*β*, regardless of preconditioning status (Figure 5(a)).

In ARDSexp, both MSCs and EVs comparably reduced protein levels of TNF-*α* and IL-6, regardless of preconditioning status. MSCs and EVs also reduced levels of KC, but levels were even lower after administration of MSCs compared to EVs (with or without serum preconditioning). VEGF levels were reduced only after administration of MSCs, independent of preconditioning status. Neither MSCs nor EVs were able to reduce TGF-*β* levels in ARDSexp, regardless of preconditioning status (Figure 5(b)).

### 3.6. Exposure to Conditioned Media or EVs from MSCs Induced Production of Anti-Inflammatory Rather Than Proinflammatory Mediators in Alveolar Macrophages *In Vitro*

**Figure 6:**
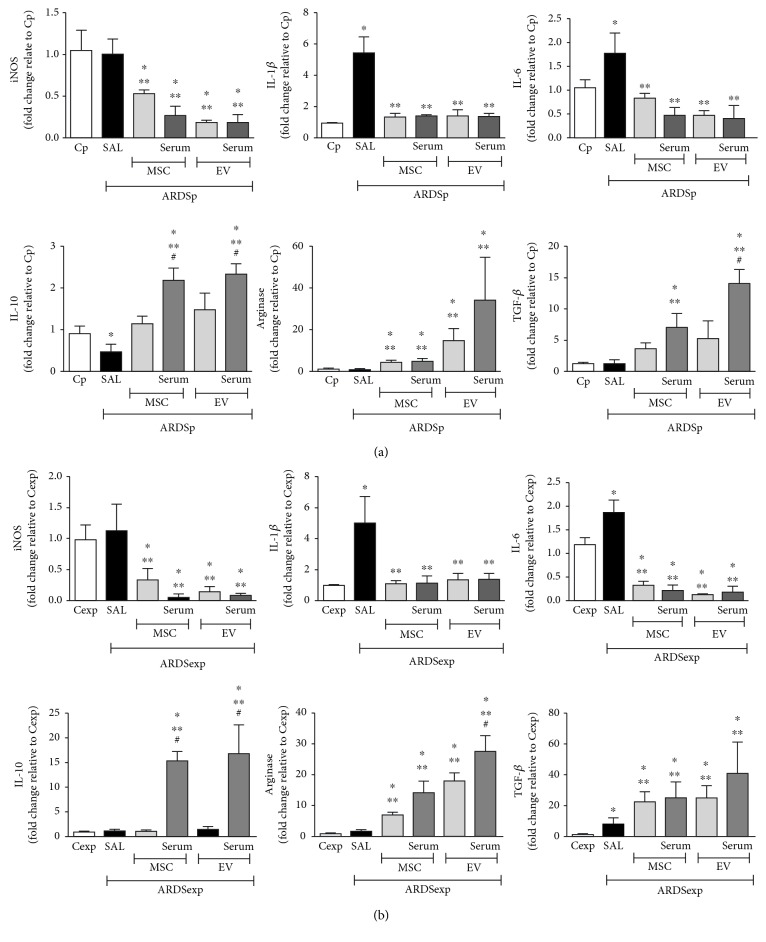
Exposure to conditioned media from MSCs or EVs induces a shift in macrophage polarization *in vitro* to the M2 rather than the M1 phenotype. Alveolar macrophages (10^5^ cells per well) were collected from (a) Cp and ARDSp or (b) Cexp and ARDSexp mice. Cells were cultured in regular conditions (Cp, Cexp, ARDSp-SAL, and ARDSexp-SAL) or with conditioned media obtained from MSCs (10^5^ cells per well) either unstimulated or stimulated with serum (serum) or extracellular vesicles derived from ARDSp or ARDSexp mice for 24 h. Relative gene expression of iNOS, IL-1*β*, IL-6, IL-10, arginase, and TGF-*β* was calculated as a ratio of average gene expression compared to expression of the housekeeping gene *36B4* and presented as fold changes relative to the Cp or Cexp group (alveolar macrophages from Cp or Cexp animals cultured with conditioned media from unstimulated MSCs). Results are presented as means + SD of alveolar macrophages pooled from 3 mice/group. All measurements were performed in triplicate. ^∗^Significantly different from the corresponding C group (*p* < 0.05). ^∗∗^Significantly different from the corresponding ARDS group (*p* < 0.05). ^#^Significantly different from the corresponding MSC group (*p* < 0.05).

Alveolar macrophages from ARDSp mice demonstrated increased expression of IL-1*β* and IL-6 and reduced expression of IL-10 compared to those from Cp animals. Exposure to conditioned media or EVs from MSCs mitigated expression of iNOS, IL-1*β*, and IL-6, regardless of preconditioning status. Conversely, alveolar macrophages demonstrated increased expression of arginase after exposure to either conditioned media or EVs from MSCs, independent of preconditioning status. Only serum-preconditioned MSCs and EVs demonstrated an increased expression of IL-10 and TGF-*β* (Figure 6(a)).

Alveolar macrophages from ARDSexp animals demonstrated increased expression of IL-1*β*, IL-6, and TGF-*β* compared to those from Cexp animals. Macrophage exposure to conditioned media or EVs from MSCs led to reductions in expression of iNOS, IL-1*β*, and IL-6, regardless of preconditioning status. On the other hand, expression of arginase and TGF-*β* increased in macrophages exposed to conditioned media or EVs from MSCs. Only serum-preconditioned MSCs and EVs demonstrated increased expression of IL-10 (Figure 6(b)).

## 4. Discussion

The primary goal of this study was to comparatively assess the effects of MSCs vs. their EVs on lung function, inflammation, and remodeling in experimental ARDSp and ARDSexp. In both ARDS groups, systemic administration of either MSCs or their EVs had several comparable effects but MSCs demonstrated even better therapeutic action, thus resulting in further improvements in lung function, histology, and inflammation. The second goal was to assess whether MSC preconditioning by exposure to serum from animals subjected to same experimental injuries could potentiate the therapeutic effects of MSCs or their EVs. Although MSC preconditioning with a biologically relevant substrate (serum from ARDS mice) yielded further expression of anti-inflammatory mediators in alveolar macrophages *in vitro*, no additional therapeutic benefit was observed in the various *in vivo* outcome measures in either ARDSp or ARDSexp.

Even though the structures primarily injured in the lungs are distinct in ARDSp (alveolar epithelium) and ARDSexp (endothelial cells), as is the underlying activation of inflammatory mechanisms [22], the models used herein induce similar impairment of lung mechanics and morphometry early in the course of lung injury [11, 27, 31, 32]. MSCs and their EVs were administered 1 day after endotoxin challenge, thus more closely resembling the situation observed in clinical practice, as changes in lung mechanics as well as inflammation and remodeling were already established. This stands in contrast with previous investigations in which MSC or EV administration was performed a few hours after injury and thus did not consider the time course of lung damage [10, 12, 27, 33]. MSCs were harvested from the bone marrow because this source has been associated with antimicrobial and anti-inflammatory effects [9, 10, 12, 34] as well as improvement in alveolar fluid clearance [35, 36], lung mechanics, gas exchange [8, 9, 11], distal organ damage [9, 37], and survival rate [10, 12, 29, 38] in different ARDS models. Furthermore, initial clinical studies of systemic administration of bone marrow-derived MSCs in patients with ARDS have shown no obvious safety issues [39, 40]. Preclinical studies have shown that EVs exert beneficial effects similar to those of MSCs. Thus, EVs have emerged as a promising therapy for testing in the clinical setting [14–17]. To facilitate comparisons across different preparations, EVs was characterized according to the criteria described by the International Society of Extracellular Vesicles [16]. Although some reports have indicated that a higher concentration of EVs would be required to obtain similar therapeutic effects as MSCs [13], we used the EV dose equivalent to the amount of MSCs administered so as to allow direct comparison of their effects and investigate whether preconditioning MSCs could lead their EVs to induce more efficient therapeutic responses.

The pathological cascade of ARDS starts with pathogen- or damage-associated molecular patterns triggering proinflammatory responses by resident airway epithelial and vascular endothelial cells. Increased secretion of TNF-*α*, IL-6, and KC not only intensifies the inflammatory process but also recruits other leukocytes (mainly neutrophils) into the lungs [41, 42]. Previous studies have indicated that MSCs induce anti-inflammatory effects on host tissue partly through paracrine actions on resident lung cells as well as inflammatory cells, with a resulting decrease in production of proinflammatory mediators [5, 8–12, 14, 15, 38]. In this context, systemic MSC administration mitigated levels of TNF-*α*, IL-6, and KC in both ARDSp and ARDSexp animals, as well as decreased inflammatory cell counts in the lungs. EVs also mitigated inflammation for most of the measured endpoints, with some exceptions. Furthermore, both conditioned media and EVs from MSCs reduced expression of proinflammatory markers (iNOS, IL-1*β*, and IL-6) in alveolar macrophages from ARDSp mice. In macrophages from ARDSexp animals, expression of proinflammatory mediators was also reduced after exposure to conditioned media or EVs from MSCs, regardless of preconditioning status. On the other hand, macrophages derived from both ARDS etiologies demonstrated higher expression of arginase, while IL-10 expression increased only in macrophages exposed to serum-preconditioned MSC-conditioned media or EVs. The effects on TGF-*β* differed according to ARDS etiology.

Increased levels of VEGF and TGF-*β* have been implicated in increased vascular permeability and fibrous proliferation in ARDS and other respiratory disorders, thereby contributing to the loss of alveolar-capillary barrier integrity [41, 43–45]. Nonetheless, cell-based therapy may stimulate TGF-*β* expression to suppress inflammatory responses [18, 21, 46–48]. In this investigation, MSCs and their EVs differently affected TGF-*β* levels *in vivo* depending on ARDS etiology. Although alveolar macrophages from ARDSp and ARDSexp demonstrated higher expression of TGF-*β* after *in vitro* exposure to conditioned media regardless of the source (MSCs and EVs), fold change in TGF-*β* expression was greater in ARDSexp than ARDSp. This suggests that different signaling pathways may be activated to induce inflammation resolution. On the other hand, only MSCs decreased the lung collagen content in both ARDSp and ARDSexp, suggesting an alternative signaling pathway for tissue repair (for example, collagenases) [11]. In addition, VEGF levels were also differently affected after MSC vs. EV administration. While MSCs significantly reduced interstitial edema, EVs were unable to mitigate this abnormality in either ARDS group. These findings contradict those of a previous study [15], which demonstrated the potential of EVs to reduce pulmonary edema in ARDSp. There are several differences in the experimental protocol that can explain these distinct results: (1) disease severity (2 vs. 4 mg·kg^−1^ of endotoxin administered intratracheally), (2) timing of EV administration after onset of lung injury (24 h vs. 12 h), and (3) route of administration (intravenous vs. intratracheal).

In fact, Zhu and collaborators [15] also found that only a modest effect was observed when the dose of EVs given for the experiments was based on the final MSC cell count; this is in agreement with our results. Therefore, they had to roughly increase the EV dose in order to enhance therapeutic effects. In the present investigation, we preconditioned cells in an attempt to potentiate EV production and release and, consequently, enhance their therapeutic effects at lower levels. MSCs were preconditioned with serum from ARDSp or ARDSexp mice because: (1) it is a biologically relevant specimen, (2) serum from patients with ARDS could be easily obtained in clinical practice, and (3) cell therapy could tailor anti-inflammatory and reparative responses according to the characteristics of ARDS in each patient. Importantly, the degree of beneficial effects after cell therapy can differ according to cell source, disease severity, etiology, and initial insult in experimental ARDS [9, 11, 25, 27, 29].

Endotoxin induced higher levels of proinflammatory and profibrotic mediators, leukocyte flow into the lungs, and pulmonary architectural distortion in ARDS mice. EV administration mitigated alveolar collapse and inflammation, confirming the therapeutic role of paracrine factors, as observed in previous reports [5, 8–12, 14, 15, 38]. Additionally, large-scale production and standardization of EVs need to be further developed in order to determine their overall therapeutic effects [13]. Nonetheless, MSC administration reduced those parameters even further while simultaneously mitigating the remodeling process, thereby improving lung function more efficiently.

## 5. Limitations

This investigation has some limitations. First, both ARDSp and ARDSexp were induced by endotoxin; therefore, these results cannot be extrapolated to other models or to the clinical scenario. Second, we used a dose of EVs equivalent to 10^5^ MSCs to allow close comparison of the different approaches evaluated herein. A higher dose of EVs might induce different effects, and a formal dose-response study might be necessary to clarify this issue. Even though the protein content within the EVs did not differ, further studies are needed to evaluate whether preconditioning modifies the nucleotide content. Finally, we performed qPCR for mRNA quantification instead of protein quantification by ELISA, since a good correlation has been observed between mRNA and protein levels of biomarkers for these models [11, 27].

## 6. Conclusion

Regardless of preconditioning, MSCs yielded greater overall improvement in ARDS, compared to EVs derived from the same number of cells. However, the effects of MSCs and EVs differed according to ARDS etiology.

## Figures and Tables

**Table 1:** Lung morphometry.

Groups		Normal alveoli (%)	Alveolar collapse (%)	Neutrophils (%)	Interstitial edema	Collagen fibers (%)
Cp		95.4 ± 1.9	4.6 ± 1.9	2.5 ± 0.8	0.17 ± 0.04	36.2 ± 2.7
ARDSp	SAL	76.6 ± 3.0^∗^	23.4 ± 3.0^∗^	14.8 ± 4.0^∗^	0.49 ± 0.10^∗^	47.7 ± 3.5^∗^
	MSC	93.2±2.1^∗∗^	6.8±2.1^∗∗^	3.0±0.4^∗∗^	0.31±0.04^∗∗^	38.7±3.1^∗∗^
	MSC serum	92.4±2.4^∗∗^	7.6±2.4^∗∗^	3.4±0.5^∗∗^	0.28±0.04^∗∗^	37.7±5.6^∗∗^
	EV	89.8±1.0^∗,∗∗^^,#^	10.2±1.0^∗,∗∗^^,#^	6.4±0.3^∗,∗∗^^,#^	0.38 ± 0.07^∗^	39.0 ± 6.2
	EV serum	86.7±2.3^∗,∗∗^^,†^	13.3±2.3^∗,∗∗^^,†^	6.5±0.7^∗,∗∗^^,†^	0.45 ± 0.15^∗^	41.3 ± 4.6

Cexp		96.4 ± 1.2	3.6 ± 1.2	2.7 ± 1.3	0.16 ± 0.07	34.3 ± 6.7
ARDSexp	SAL	73.4 ± 5.4^∗^	26.6 ± 5.4^∗^	21.3 ± 2.3^∗^	0.44 ± 0.08^∗^	43.9 ± 3.4^∗^
	MSC	92.0±2.7^∗∗^	8.0±2.7^∗∗^	5.1±0.5^∗∗^	0.25±0.07^∗∗^	37.5±2.3^∗∗^
	MSC serum	93.3±1.9^∗∗^	6.7±1.9^∗∗^	4.3±1.1^∗∗^	0.29±0.06^∗∗^	37.8±1.8^∗∗^
	EV	88.7±1.6^∗,∗∗^^,#^	11.3±1.6^∗,∗∗^^,#^	7.8±0.4^∗,∗∗^^,#^	0.30 ± 0.07^∗^	40.8 ± 1.6
	EV serum	87.6±2.9^∗,∗∗^^,†^	12.4±2.9^∗,∗∗^^,†^	8.8±1.7^∗,∗∗^^,†^	0.39 ± 0.09^∗^	42.9 ± 6.1

Fraction area of normal and collapsed alveoli, neutrophil cell count, interstitial edema, and collagen fiber content in the alveolar septa. All values were computed in 10 random, noncoincident fields of view per mouse. Values were expressed as mean ± standard deviation of six animals per group. ARDS was induced by administration of *Escherichia coli* lipopolysaccharide intratracheally (ARDSp) or intraperitoneally (ARDSexp). Control mice (C) received saline solution intratracheally (Cp) or intraperitoneally (Cexp). After 24 h, ARDSp and ARDSexp animals were further randomized to receive saline (50 *μ*L, SAL), bone marrow-derived MSCs (10^5^, 50 *μ*L), or EVs (10^5^, 50 *μ*L), stimulated (MSC serum, EV serum) or not with serum (MSCs, EVs) obtained from ARDSp or ARDSexp animals. ^∗^Significantly different from the corresponding C group. ^∗∗^Significantly different from the corresponding ARDS group. ^#^Significantly different from MSC. ^†^Significantly different from MSC serum (*p* < 0.05).

## Data Availability

The data used to support the findings of this study are available from the corresponding author upon request.

## Supplementary Materials

Supplementary materials Supplementary Table 1 PCR primer sequences. Supplementary Fig. 1: characterization of EVs. Supplementary Fig. 2: total protein content of extracellular vesicles (EVs), particle size, and EV concentration, measured using the Bradford assay and nanoparticle tracking analysis.
