# Supplementary material for: Mesenchymal Stromal Cells Are More Effective Than Their Extracellular Vesicles at Reducing Lung Injury Regardless of Acute Respiratory Distress Syndrome Etiology
Source: Stem Cells Int. 2019 Aug 21;2019:8262849. doi: 10.1155/2019/8262849 (PMC6720722; doi:10.1155/2019/8262849)
Supplement: Supplementary materials — Supplementary Table 1 PCR primer sequences. Supplementary Fig. 1: characterization of EVs. Supplementary Fig. 2: total protein content of extracellular vesicles (EVs), particle size, and EV concentration, measured using the Bradford assay and nanoparticle tracking analysis. [file 8262849.f1.docx]

**Supplementary Table 1**

**Supplementary Table 1 –** PCR primer sequences

| Gene | Forward | Reverse |
| --- | --- | --- |
| Arginase | 5′-GCT CAG GTG AAT CGG CCT TTT-3′ | 5′-TGG CTT GCG AGA CGT AGA C-3′ |
| TGF-β1 | 5′-TGA CGT CAC TGG AGT TGT ACG G-3′ | 5′-GGT TCA TGT CAT GGA TGG TGC-3′ |
| IL-10 | 5′-ATC CAA GAC AAC ACT ACT ATA-3′ | 5′-TAA ATA TCC TCA AAG TTC C-3’ |
| iNOS | 5′-CTT CAG GTA TGC GGT ATT GG-3′ | 5′-CAT GGT GAA CAC GTT CTT GG-3′ |
| IL-1β | 5′-GTT GAC GGA CCC CAA AAG-3′ | 5′- GTG CTG CTG CGA GAT TTG - 3′ |
| IL-6 | 5′-GGC TAC CAT GCC AAC TTC T-3′ | 5′-TGT ACA ACC AGC ATA ACC CGG-3′ |
| 36B4 | 5′-CAA CCCAGC TCT GGA GAA AC-3′ | 5′-GTT CTG AGC TGG CAC AGT GA-3′ |

TGF, transforming growth factor; IL, interleukin; iNOS, inducible nitric oxide synthase; 36B4, acidic ribosomal phosphoprotein P0.


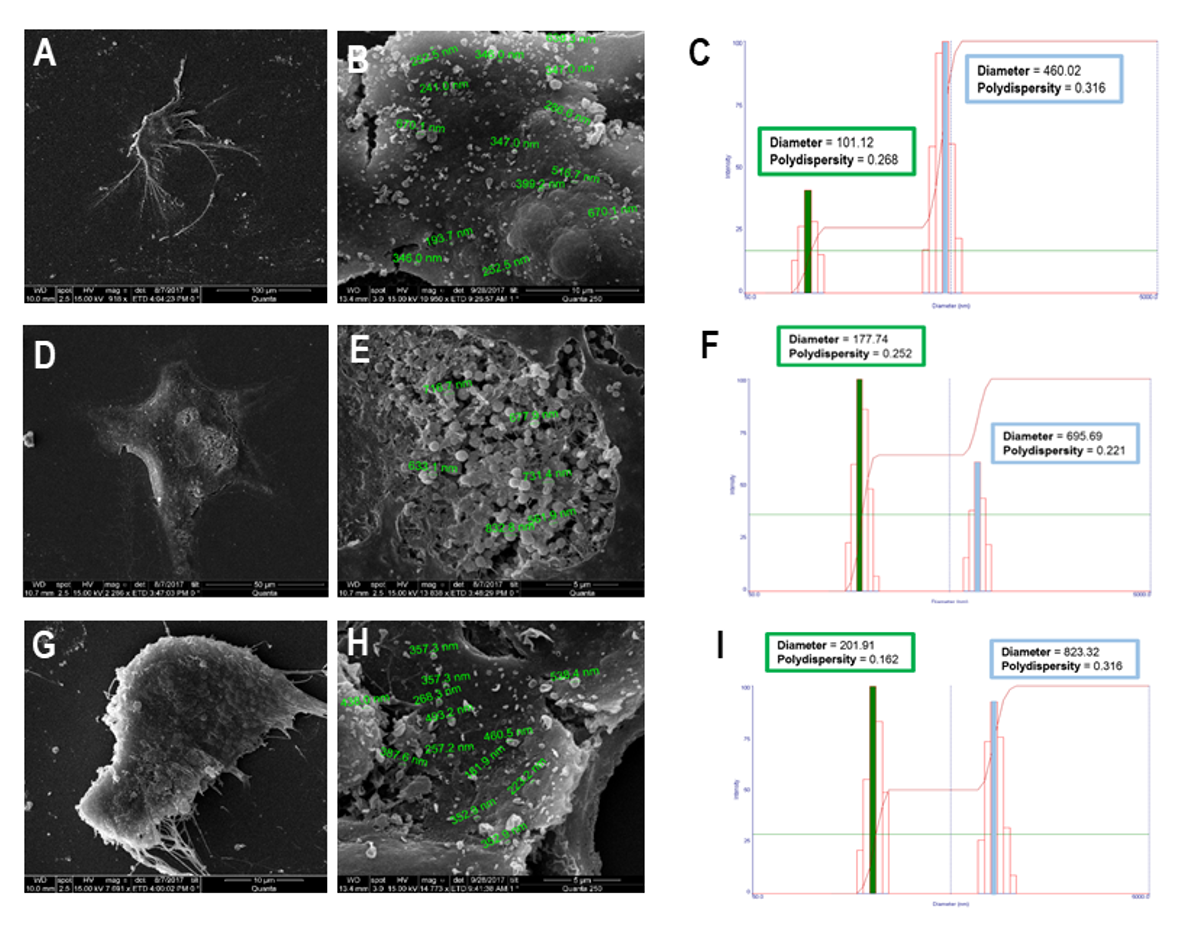


**Supplementary Fig. 1 – Characterization of EVs.** Scanning electron microscopy of bone marrow-derived mesenchymal stromal cells (MSCs). **A-B**: MSCs before induction of cellular stress (fetal bovine serum deprivation), showing the presence of extracellular vesicles. **D-E**: MSCs after cellular stress induction with serum obtained from animals subjected to pulmonary acute respiratory distress syndrome (ARDSp), showing an increase in the number of vesicles on the cell surface. **G-H:**  MSCs after cellular stress induction with serum obtained from subjected to extrapulmonary acute respiratory distress syndrome (ARDSexp), showing an increase in the number of vesicles on the cell surface. **C, F, I:** Representative graphs of the intensity and hydrodynamic diameter of extracellular vesicle samples analyzed using the dynamic light scattering technique. Graphs show two populations of extracellular vesicles obtained from MSCs (C), MSCs stimulated with serum obtained from ARDSp animals (F), and MSCs stimulated with serum obtained from ARDSexp animals (I); one of lower intensity and medium size, characteristic of exosomes, and another with greater intensity and average size, characteristic of microvesicles.

**
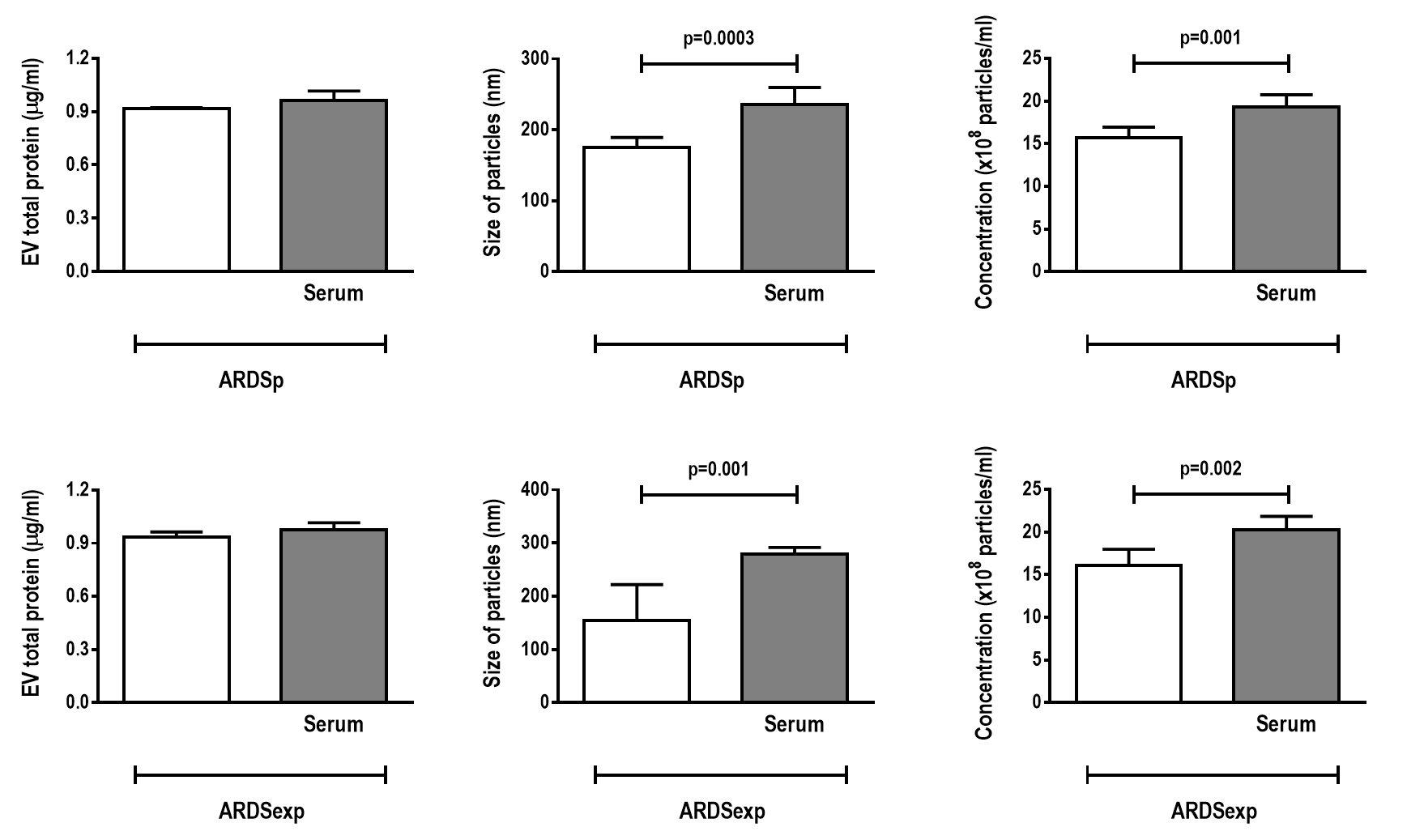
**

**Supplementary Fig. 2 – Total protein content of extracellular vesicles (EVs), particle size, and EV concentration, measured using the Bradford assay and nanoparticle tracking analysis, respectively.** Analyses done before and after MSC cellular stress induction with serum obtained from animals subjected to pulmonary (ARDSp) and extrapulmonary (ARDSexp) acute respiratory distress syndrome. Data are expressed as mean (SD) of 4-5 samples in each group.
